# Sociodemographic Factors of Asthma Prevalence and Costs Among Children and Adolescents in the United States, 2016–2021

**DOI:** 10.5888/pcd21.230449

**Published:** 2024-07-25

**Authors:** Nianyang Wang, Tursynbek Nurmagambetov

**Affiliations:** 1 Centers for Disease Control and Prevention, National Center for Environmental Health, Division of Environmental Health Science and Practice, Asthma and Air Quality Branch, Atlanta, Georgia

## Abstract

**Introduction:**

Asthma is a chronic condition with a high prevalence and cost of care among children and adolescents. While previous research described the association of sociodemographic factors with childhood asthma prevalence, there is limited knowledge of these factors’ association with medical expenditures. In this study, we examined disparities in treated asthma prevalence and medical expenditures among US children and adolescents.

**Methods:**

Using nationally representative data from the 2016–2021 Medical Expenditures Panel Survey, we conducted a cross-sectional study of 2,365 children and adolescents (aged 0–17 y) with treated asthma compared with 40,497 children and adolescents without treated asthma. Treated asthma was defined as whether the child or adolescent had a medical event (emergency department visit, hospital inpatient stay, hospital outpatient visit, office-based medical visit, home health, and/or prescribed medicines) due to asthma. We controlled for sociodemographic factors of race and ethnicity, age, sex, health insurance coverage, family poverty status, and census region. We used 2-part models and generalized linear models to estimate annual per-person incremental medical expenditures associated with asthma.

**Results:**

Children and adolescents with treated asthma were more likely than those without treated asthma to be non-Hispanic Black or Hispanic, male, and publicly insured. Children and adolescents with treated asthma had $3,362.56 in additional annual medical expenditures, of which $174.06 was out-of-pocket, compared with children and adolescents without treated asthma. The additional expenditures included $955.96 for prescribed medicines, $151.52 for emergency department visits, and $858.17 for office-based medical visits. Non-Hispanic Black children with treated asthma had significantly lower total ($2,721.28) and office-based visit expenditures ($803.19) than non-Hispanic White children with treated asthma.

**Conclusion:**

Disparities among children and adolescents in the US persist in treated asthma prevalence and associated medical expenditures by sociodemographic factors.

**Summary**

**What is already known on this topic?**

Sociodemographic factors such as race and ethnicity are associated with the prevalence of asthma in children and adolescents.

**What is added by this report?**

While non-Hispanic Black children and adolescents had a higher prevalence of asthma than non-Hispanic White children and adolescents, non-Hispanic Black children and adolescents with treated asthma had $2,721.28 lower total medical expenditures and $803.19 lower office-based visit expenditures than non-Hispanic White children and adolescents with treated asthma.

**What are the implications for public health practice?**

This research can help public health practitioners direct additional attention and resources to reduce racial and ethnic disparities in the prevalence and cost of asthma among children and adolescents.

## Introduction

Asthma is a chronic respiratory condition characterized by inflammation of the airways, leading to recurrent episodes of wheezing, breathlessness, chest tightness, and coughing. Asthma can affect people of all ages, but it often starts in childhood (1). It is one of the most prevalent and costly chronic conditions among children and adolescents and currently affects more than 4.6 million children and adolescents in the US (2). In 2013, the direct cost of pediatric asthma in the US was $5.92 billion; children and adolescents with asthma used more health care than those without asthma (3). Previous research using 2008–2013 national data estimated that children and adolescents who seek treatment for asthma have an additional $1,737 in annual medical expenditures compared with children and adolescents without asthma (4).

Several sociodemographic factors, such as race and ethnicity and family income, are associated with the prevalence of asthma among children and adolescents (5). Non-Hispanic Black (hereinafter, Black) children and adolescents are more than twice as likely than non-Hispanic White (hereinafter, White) children and adolescents to have asthma (11.6% vs 5.5%), and people below 100% of the federal poverty threshold are significantly more likely than people at or above 450% of the poverty threshold to have asthma (10.4% vs 6.8%) (2). Previous studies showed significantly lower medical expenditures among Black children and adolescents than among White children and adolescents and no significant differences in medical expenditures among different income levels (6,7).

Asthma management consists of a broad range of tools that include environmental factors, medication, and health education; however, the role of sociodemographic factors such as race and ethnicity as a marker of social factors that influence incremental medical expenditures for children with treated asthma has not been well examined (8). In this study, we examined the sociodemographic factors surrounding treated asthma in children, defined herein as persons aged 0 to 17 years, and quantified the per-person medical expenditures associated with treated asthma. We also hypothesized that racial and ethnic minority children, compared with White children, would have a higher prevalence of treated asthma and lower incremental expenditures of treated asthma.

## Methods

We performed a pooled cross-sectional analysis of data from the 2016–2021 Medical Expenditures Panel Survey (MEPS) (9). MEPS is conducted annually from a subsample of National Health Interview Survey households to obtain detailed data on the medical expenditures of the noninstitutionalized US civilian population. We restricted our sample to children aged 0 to 17 years. First, we used the full-year Household Component of MEPS to obtain our sociodemographic factors of interest and expenditures data. We then combined the data from the Household Component with the relevant survey’s Medical Event files to examine emergency department visits, hospital inpatient stays, hospital outpatient visits, office-based medical visits, home health visits, and prescribed medicines use for each respondent. We used the Medical Conditions file to establish that the medical event was due to asthma. MEPS’s full-year response rate ranged from 46.0% in 2016 to 21.8% in 2021 (10). We pooled multiple years of data and used the variance linkage file (HC-036) to link data. We also used the complex survey design to create nationally representative estimates for US children. We adjusted all types of expenditures to the corresponding medical price index in 2021 US dollars (11).

### Measures

The main outcomes of this analysis were treated asthma and associated medical expenditures. Treated asthma in children was defined by whether a child had at least 1 medical event (emergency department visit, hospital inpatient stay, hospital outpatient visit, office-based medical visit, home health, or prescribed medicine) in the calendar year that was due to asthma based on the Clinical Classifications Software Refined (CCSR) diagnosis code for asthma (RESP009) in the Medical Conditions file and was linked to an event in the Medical Event file (12). In accordance with MEPS analysis guidelines, we excluded informal telephone calls that came from office-based medical visits and hospital outpatient events and informal home health visits from our definition of treated asthma because these events are not associated with treating asthma (13). Starting in 2020, MEPS began including telehealth events that were classified as office-based medical visits or hospital outpatient events, and telehealth events that were used to treat asthma were included in our definition of treated asthma (13). MEPS also has a medical event category of “other medical equipment and services”; however, we excluded this category because it is not linked to the Medical Conditions file (13).

We measured total medical expenditures as the combined medical expenditures for each medical event category (emergency department visit, hospital inpatient stay, hospital outpatient visit, office-based medical visit, home health, and prescribed medicine) in the calendar year for the child/adolescent. We used 4 perspectives for this study: family or out-of-pocket, public payer, private payer, and all possible payers combined. We defined out-of-pocket medical expenditures as the total medical expenditures for the calendar year that were paid out-of-pocket for the medical care of the respondent. We also examined the total expenditures for each type of medical event (emergency department visit, hospital inpatient stay, hospital outpatient visit, office-based medical visit, home health, and prescribed medicine) during the whole calendar year.

### Sociodemographic factors

We controlled for the sociodemographic factors of race and ethnicity, age, sex, family income, health insurance coverage, and region. Data on race and ethnicity were self-reported and coded as Hispanic; non-Hispanic Asian, Native Hawaiian, or Pacific Islander only (Asian); non-Hispanic Black only (Black), non-Hispanic White only (White), and non-Hispanic Other race, which includes American Indian, Alaska Native, and multiracial (Other). We categorized age into the following groups: 0 to 4 years, 5 to 14 years, and 15 to 17 years. Sex was defined as male or female. Family poverty status was determined by total family income divided by the applicable federal poverty line based on family size and composition and classified as negative or poor (≤100%), near poor (>100%–125%), low income (>125%–200%), middle income (>200%–400%), and high income (>400%). Health insurance coverage included the following categories: covered by private insurance (private insurance and TRICARE) at any time in the calendar year, covered only by public insurance (Medicaid and Medicare) during the calendar year, or uninsured for the entire calendar year. The regions were defined as Midwest, Northeast, South, and West. Our final sample consisted of 2,365 children (3,697,530 weighted) with treated asthma and 40,497 children (69,795,477 weighted) without treated asthma. This study was exempt from institutional review board review because it was a secondary data analysis of deidentified survey data.

### Statistical analysis

We first summarized the descriptive statistics of our sample according to treated asthma status (children with treated asthma vs children without treated asthma); we used χ^2^ tests to determine significant differences between the 2 groups. We used a multivariable logistic regression to estimate the association of sociodemographic factors with treated asthma as the outcome, and we calculated odds ratios (ORs) and 95% CIs. We used a 2-part model to estimate total all-payers medical expenditures, total out-of-pocket medical expenditures, total private payer medical expenditures, total public payer medical expenditures, and total medical expenditures for each medical event category. The model shows incremental medical expenditures for treated asthma and race and ethnicity while controlling for all other sociodemographic variables. The 2-part model was necessary because expenditures data are skewed to the left, are heteroskedastic, and have a large proportion of people with $0 in annual medical expenditures. To estimate incremental medical expenditures, we used the “twopm” command in Stata (StataCorp LLC). The first part of the 2-part model was a logit model, and the second part was a generalized linear regression with a log link and a gamma distribution. After running this model, we applied marginal effects to the independent variable of interest to determine its marginal or discrete effect on the continuous or discrete dependent variable, respectively (14). Finally, we used a 2-part model again to analyze the incremental medical event expenditures of treated asthma by each year. All analyses were completed in Stata version 18; significance was set at the .05 level.

## Results

Among all races and ethnicities, Black children comprised a larger proportion of children in the population with treated asthma (21.8%) than in the population without treated asthma (13.2%) (*P* < .001) (Table 1). Among age groups, children with treated asthma were less likely to be aged 0 to 4 years (14.8%) than children without treated asthma (27.1%) (*P* < .001). Among children from families with negative or poor incomes, the percentage of children with treated asthma (22.7%) was higher than the percentage of children without treated asthma (16.4%) (*P* < .001). Children with treated asthma were more likely to be male than female (58.9% vs 41.1%), while the difference was smaller in children without treated asthma (50.6% vs 49.4%) (*P* < .001). Children with treated asthma were more likely than children without treated asthma to have public insurance (48.1% vs 36.6%) (*P* < .001) and to reside in the Northeast (19.6% vs 15.7%) and Midwest (24.6% vs 20.9%) (*P* = .002). Finally, treated asthma was more common in the first 3 years of our data (18.4% in 2016, 18.7% in 2017, 19.1% in 2018) than in the last 3 years (16.7% in 2019, 14.2% in 2020, 12.9% in 2021) (*P* < .001).

**Table 1:** Weighted Sample Characteristics of US Children and Adolescents, by Treated Asthma Status, 2016–2021^a^

Characteristic	Children without treated asthma, %	Children with treated asthma, %	Unweighted no. (%)	*P* value^b^
**Race and ethnicity**				
Hispanic	25.4	27.0	15,447 (25.5)	<.001
Non-Hispanic Asian	5.4	3.7	2,094 (5.3)	
Non-Hispanic Black	13.2	21.8	7,026 (13.7)	
Non-Hispanic White	49.8	41.0	15,688 (49.4)	
Non-Hispanic Other^c^	6.2	6.7	2,607 (6.2)	
**Age, y**				
0-4	27.1	14.8	10,303 (26.5)	<.001
5-14	55.2	66.1	24,977 (55.7)	
15–17	17.7	19.1	7,582 (17.8)	
**Sex**				
Male	50.6	58.9	21,969 (51.0)	<.001
Female	49.4	41.1	20,893 (49.0)	
**Health insurance coverage**				
Any private	60.6	50.4	19,955 (60.1)	<.001
Public only	36.6	48.1	21,508 (37.2)	
Uninsured	2.8	1.4	1,399 (2.7)	
**Family poverty status^d^**				
Negative or poor^e^	16.4	22.7	11,905 (16.7)	<.001
Near poor	5.5	7.4	2,996 (5.6)	
Low income	15.0	18.0	7,294 (15.2)	
Middle income	30.0	24.7	11,203 (29.7)	
High income	33.1	27.2	9,464 (32.8)	
**Region**				
Northeast	15.7	19.6	5,968 (15.9)	.002
Midwest	20.9	24.6	8,607 (21.1)	
South	39.0	35.1	17,000 (38.8)	
West	24.3	20.7	11,287 (24.2)	
**Year**				
2016	16.7	18.4	9,211 (16.8)	<.001
2017	16.6	18.7	8,158 (16.7)	
2018	16.6	19.1	7,449 (16.7)	
2019	16.8	16.7	6,558 (16.8)	
2020	16.6	14.2	5,929 (16.4)	
2021	16.8	12.9	5,557 (16.6)	

^a^ Data source: 2016–2021 Medical Expenditures Panel Survey. All estimates are pooled and weighted, adjusting for the complex survey design.

^b^ Pearson χ^2^ test used to test differences.

^c^ Includes American Indian, Alaska Native, and multiracial.

^d^ Total family income is divided by the applicable federal poverty line based on family size and composition and classified as negative or poor (≤100%), near poor (>100%–125%), low income (>125%–200%), middle income (>200%–400%), or high income (>400%).

^e^ Negative income is when an individual's total expenses exceed their total income.

In the multivariable logistic regression of the sociodemographic factors affecting treated asthma status, children with treated asthma were more likely to be Black (OR = 1.78; 95% CI, 1.45–2.19) or Hispanic (OR = 1.22; 95% CI, 1.01–1.48) than non-Hispanic White, aged 5 to 14 years (OR = 2.22; 95% CI, 1.83–2.68) or 15 to 17 years (OR = 2.05; 95% CI, 1.61–2.59) than aged 0 to 4 years, male (OR = 1.40; 95% CI, 1.22–1.61) than female, publicly insured (OR = 1.28; 95% CI, 1.06–1.54) than privately insured, and from the Midwest (OR = 1.51; 95% CI, 1.21–1.89) or Northeast (OR = 1.47; 95% CI, 1.18–1.83) than from the South, and less likely to have no health insurance than have private insurance (OR = 0.55; 95% CI, 0.32–0.96) (Table 2).

**Table 2:** Multivariable Logistic Regression of Factors Influencing Children’s and Adolescents’ Treated Asthma Status^a^

Characteristic	Odds Ratio (95% CI) [*P* value]
**Race and ethnicity**	
Hispanic	1.22 (1.01–1.48) [.04]
Non-Hispanic Asian	0.82 (0.52–1.30) [.40]
Non-Hispanic Black	1.78 (1.45–2.19) [<.001]
Non-Hispanic White	1 [Reference]
Non-Hispanic Other^b^	1.29 (0.96–1.72) [.09]
**Age, y**	
0–4	1 [Reference]
5–14	2.22 (1.83–2.68) [<.001]
15–17	2.05 (1.61–2.59) [<.001]
**Sex**	
Male	1.40 (1.22–1.61) [<.001]
Female	1 [Reference]
**Health insurance coverage**	
Any private	1 [Reference]
Public only	1.28 (1.06–1.54) [.01]
Uninsured	0.55 (0.32–0.96) [.04]
**Family income**	
Negative or poor^c^	1.25 (0.96–1.62) [.10]
Near poor	1.24 (0.91–1.70) [.16]
Low income	1.17 (0.90–1.52) [.24]
Middle income	0.89 (0.73–1.09) [.26]
High income	1 [Reference]
**Region**	
Midwest	1.51 (1.21–1.89) [<.001]
Northeast	1.47 (1.18–1.83) [.001]
West	1.03 (0.83–1.27) [.80]
South	1 [Reference]

^a^ Data source: 2016–2021 Medical Expenditures Panel Survey. All estimates are pooled and weighted, adjusting for the complex survey design.

^b^ Includes American Indian, Alaska Native, and multiracial.

^c^ Negative income is when an individual’s total expenses exceed their total income.

The 2-part model shows that children with treated asthma had additional expenditures for all categories of medical events (Table 3). Children with treated asthma had $3,362.56 (95% CI, $2,654.55 to $4,070.57) in additional annual total medical expenditures, while Black children had $1,256.50 lower (95% CI, −$1,578.66 to −$934.34) total medical expenditures and Hispanic children had $785.34 lower (95% CI, −$1,140.21 to −$430.47) total medical expenditures than White children. Children with treated asthma had $174.06 (95% CI, $81.39 to $266.73) in additional annual out-of-pocket medical expenditures, while Black children had $226.86 lower (95% CI, −$265.47 to −$188.25) out-of-pocket medical expenditures and Hispanic children had $106.66 lower (95% CI, −$153.44 to −$59.88) out-of-pocket medical expenditures than White children. Children with treated asthma had $955.96 in additional medical expenditures for prescribed medicine (95% CI, $776.99 to $1,134.93) than children without treated asthma, while Black children and Hispanic children had lower medical expenditures for prescribed medicines (−$155.80, 95% CI, −$217.90 to −$93.71; and −$142.02, 95% CI, −$201.12 to −$82.93, respectively) than White children. In addition, children with treated asthma had $858.17 in additional medical expenditures for office-based medical visits (95% CI, $613.85 to $1,102.50) than children without treated asthma while Black children and Hispanic children had lower medical expenditures for office-based medical visits (−$467.25; 95% CI, −$559.46 to −$375.04 and −$172.18; 95% CI, −$287.67 to −$56.68, respectively) than White children. Finally, children with treated asthma had $151.52 in additional medical expenditures for emergency department visits (95% CI, $101.76 to $201.27).

**Table 3:** Two-Part Model of Incremental Medical Expenditures of US Children, by Race and Ethnicity and Treated Asthma, 2016–2021^a^

Characteristic	Logit estimate (95% CI)	*P* value	GLM estimate (95% CI), $	*P* value
**Total**				
Treated asthma status				
With treated asthma	—	—	3,362.56 (2,654.55 to 4,070.57)	<.001
Without treated asthma	—	—	Reference	
Race and ethnicity				
Hispanic	—	—	−785.34 (−1,140.21 to −430.47)	<.001
Non-Hispanic Asian	—	—	−1,376.94 (−1,776.66 to −977.22)	<.001
Non-Hispanic Black	—	—	−1,256.50 (−1,578.66 to −934.34)	<.001
Non-Hispanic White	—	—	Reference	
Non-Hispanic Other^b^	—	—	−400.81 (−954.00 to 152.38)	.16
**Total private payer**				
Treated asthma status				
With treated asthma	1.189 (0.963 to 1.415)	<.001	1,188.74 (597.02 to 1,780.46)	<.001
Without treated asthma	Reference		Reference	
Race and ethnicity				
Hispanic	−0.525 (−0.677 to −0.372)	<.001	−233.54 (−501.20 to 34.12)	.09
Non-Hispanic Asian	−0.536 (−0.813 to −0.260)	<.001	−600.90 (−882.70 to −319.10)	<.001
Non-Hispanic Black	−0.703 (−0.887 to −0.518)	<.001	−560.58 (−800.15 to −321.01)	<.001
Non-Hispanic White	Reference		Reference	
Non-Hispanic Other^b^	−0.486 (−0.721 to −0.250)	<.001	−230.90 (−584.70 to 122.89)	.20
**Total public payer**				
Treated asthma status				
With treated asthma	1.445 (1.279 to 1.611)	<.001	1,499.60 (1,135.31 to 1,863.90)	<.001
Without treated asthma	Reference		Reference	
Race and ethnicity				
Hispanic	−0.096 (−0.236 to 0.044)	.18	−270.01 (−433.87 to −106.15)	.001
Non-Hispanic Asian	−0.403 (−0.718 to −0.087)	.01	−705.02 (−862.59 to −547.46)	<.001
Non-Hispanic Black	−0.114 (−0.325 to 0.096)	.29	−382.74 (−551.55 to −213.92)	<.001
Non-Hispanic White	Reference		Reference	—
Non-Hispanic Other^b^	0.039 (−0.190 to 0.269)	.74	−120.81 (−423.73 to 182.10)	.43
**Total out-of-pocket**				
Treated asthma status				
With treated asthma	1.378 (1.236 to 1.521)	<.001	174.06 (81.39 to 266.73)	<.001
Without treated asthma	Reference		Reference	
Race and ethnicity				
Hispanic	−0.398 (−0.482 to −0.314)	<.001	−106.66 (−153.44 to −59.88)	<.001
Non-Hispanic Asian	−0.350 (−0.536 to −0.164)	<.001	−98.09 (−173.51 to −22.66)	.01
Non-Hispanic Black	−0.698 (−0.818 to −0.579)	<.001	−226.86 (−265.47 to −188.25)	<.001
Non-Hispanic White	Reference		Reference	
Non-Hispanic Other^b^	−0.365 (−0.505 to −0.224)	<.001	−130.46 (−188.55 to −72.37)	<.001
**Prescribed medicine**				
Treated asthma status				
With treated asthma	4.468 (4.000 to 4.936)	<.001	955.96 (776.99 to 1,134.93)	<.001
Without treated asthma	Reference		Reference	
Race and ethnicity				
Hispanic	−0.392 (−0.490 to −0.294)	<.001	−142.02 (−201.12 to −82.93)	<.001
Non-Hispanic Asian	−0.839 (−1.003 to −0.675)	<.001	−256.41 (−307.71 to −205.10)	<.001
Non-Hispanic Black	−0.631 (−0.752 to −0.511)	<.001	−155.80 (−217.90 to −93.71)	<.001
Non-Hispanic White	Reference		Reference	
Non-Hispanic Other^b^	−0.258 (−0.444 to −0.073)	<.001	−47.89 (−137.68 to 41.91)	.30
**Office-based medical visits**				
Treated asthma status				
With treated asthma	1.205 (1.026 to 1.384)	<.001	858.17 (613.85 to 1,102.50)	<.001
Without treated asthma	Reference		Reference	
Race and ethnicity				
Hispanic	−0.470 (−0.582 to −0.359)	<.001	−172.18 (−287.67 to −56.68)	.004
Non-Hispanic Asian	−0.521 (−0.709 to −0.333)	<.001	−406.62 (−521.22 to −292.02)	<.001
Non-Hispanic Black	−0.734 (−0.856 to −0.613)	<.001	−467.25 (−559.46 to −375.04)	<.001
Non-Hispanic White	Reference		Reference	
Non-Hispanic Other^b^	−0.368 (−0.564 to −0.172)	<.001	−216.08 (−331.89 to −100.28)	<.001
**Emergency department visits**				
Treated asthma status				
With treated asthma	0.918 (0.751 to 1.085)	<.001	151.52 (101.76 to 201.27)	<.001
Without treated asthma	Reference		Reference	
Race and ethnicity				
Hispanic	−0.071 (−0.198 to 0.056)	.27	−13.45 (−32.03 to 5.12)	.16
Non-Hispanic Asian	−0.420 (−0.678 to −0.162)	<.001	−47.35 (−70.12 to −24.59)	<.001
Non-Hispanic Black	−0.043 (−0.203 to 0.117)	.60	−17.63 (−38.78 to 3.52)	.10
Non-Hispanic White	Reference		Reference	
Non-Hispanic Other^b^	0.137 (−0.066 to 0.340)	.19	4.58 (−26.01 to 35.17)	.77
**Inpatient stays**				
Treated asthma status				
With treated asthma	1.244 (0.957 to 1.531)	<.001	685.39 (291.11 to 1,079.67)	.001
Without treated asthma	Reference		Reference	
Race and ethnicity				
Hispanic	−0.201 (−0.422 to 0.020)	.07	−205.46 (−401.31 to −9.62)	.04
Non-Hispanic Asian	−0.716 (−1.261 to −0.170)	.01	−386.02 (−628.81 to −143.22)	.002
Non-Hispanic Black	−0.265 (−0.527 to −0.004)	.046	−72.73 (−304.04 to 158.57)	.54
Non-Hispanic White	Reference		Reference	
Non-Hispanic Other^b^	0.220 (−0.109 to 0.549)	.19	−84.91 (−360.70 to 190.88)	.55
**Hospital outpatient**				
Treated asthma status				
With treated asthma	0.686 (0.518 to 0.855)	<.001	243.22 (66.15 to 420.30)	.007
Without treated asthma	Reference		Reference	
Race and ethnicity				
Hispanic	−0.326 (−0.498 to −0.155)	<.001	−89.82 (−152.11 to −27.54)	.005
Non-Hispanic Asian	−0.231 (−0.585 to 0.123)	.20	−122.60 (−229.28 to −15.92)	.02
Non-Hispanic Black	−0.696 (−0.896 to −0.497)	<.001	−135.35 (−189.84 to −80.86)	<.001
Non-Hispanic White	Reference		Reference	
Non-Hispanic Other^b^	−0.175 (−0.416 to 0.066)	.15	−117.63 (−191.95 to −43.31)	.002
**Home health**				
Treated asthma status				
With treated asthma	0.879 (0.515 to 1.244)	<.001	99.34 (−5.21 to 203.90)	.06
Without treated asthma	Reference		Reference	
Race and ethnicity				
Hispanic	−0.385 (−0.744 to −0.026)	.04	87.52 (6.47 to 168.58)	.03
Non-Hispanic Asian	−0.598 (−1.313 to 0.118)	.10	−14.95 (−101.39 to 71.49)	.73
Non-Hispanic Black	−0.593 (−1.027 to −0.158)	.008	−12.12 (−91.00 to 66.77)	.76
Non-Hispanic White	Reference		Reference	
Non-Hispanic Other^b^	0.204 (−0.318 to 0.727)	.44	69.57 (−58.42 to 197.56)	.29

Abbreviation: GLM, generalized linear model.

^a^ Data source: 2016–2021 Medical Expenditures Panel Survey. All estimates were adjusted for the sociodemographic factors included in the analysis. All estimates are pooled and weighted, adjusting for the complex survey design. Expenditures are adjusted to 2021 US dollars.

^b^ Includes American Indian, Alaska Native, and multiracial.

The incremental total, prescribed medicines, office-based medical visits, and emergency department visits medical expenditures for treated asthma was positive and high for all years, while incremental out-of-pocket medical expenditures were not significantly different from 0 in 2020 and 2021 (Table 4).

**Table 4:** US Annual Per-Child Incremental Medical Expenditures of Treated Asthma, 2016–2021^a^

Category	Estimate (95% CI), $	*P* value^b^
**Total**		
Pooled	3,362.56 (2,654.55 to 4,070.57)	<.001
2016	3,016.90 (1,864.87 to 4,168.94)	<.001
2017	4,327.59 (2,609.10 to 6,046.08)	<.001
2018	3,687.54 (2,387.72 to 4,987.35)	<.001
2019	3,085.89 (1,894.30 to 4,277.48)	<.001
2020	3,257.84 (1,213.67 to 5,302.01)	.002
2021	3,049.68 (1,419.40 to 4,679.96)	<.001
**Total private payer**		
Pooled	1,188.74 (597.02 to 1,780.46)	<.001
2016	1,260.57 (337.14 to 2,184.00)	.008
2017	3,360.82 (959.22 to 5,762.42)	.006
2018	4,088.02 (1832.01 to 6,344.02)	<.001
2019	871.83 (81.25 to 1,662.42)	.03
2020	649.17 (−630.26 to 1,928.60)	.32
2021	19.81 (−420.62 to 460.24)	.93
**Total public payer**		
Pooled	1,499.60 (1,135.31 to 1,863.90)	<.001
2016	1,639.72 (903.30 to 2,376.13)	<.001
2017	2,177.15 (945.44 to 3,408.85)	.001
2018	948.75 (578.72 to 1,318.78)	<.001
2019	1,571.21 (793.35 to 2,349.07)	<.001
2020	1,641.26 (704.41 to 2,578.11)	.001
2021	2,142.29 (1,094.65 to 3,189.93)	<.001
**Total out-of-pocket**		
Pooled	174.06 (81.39 to 266.73)	<.001
2016	195.82 (72.92 to 318.72)	.002
2017	199.68 (78.14 to 321.22)	.001
2018	368.12 (5.14 to 731.11)	.047
2019	224.44 (52.42 to 396.46)	.01
2020	10.28 (−87.95 to 108.51)	.84
2021	129.60 (−94.31 to 353.50)	.26
**Prescribed medicine**		
Pooled	955.96 (776.99 to 1,134.93)	<.001
2016	854.74 (609.15 to 1,100.33)	<.001
2017	1,378.30 (838.16 to 1,918.44)	<.001
2018	1,161.92 (757.98 to 1,565.86)	<.001
2019	957.07 (650.21 to 1,263.94)	<.001
2020	633.51 (398.85 to 868.18)	<.001
2021	1,006.50 (598.91 to 1,414.10)	<.001
**Office-based medical visits**		
Pooled	858.17 (613.85 to 1,102.50)	<.001
2016	791.53 (413.46 to 1,169.60)	<.001
2017	989.58 (570.86 to 1,408.29)	<.001
2018	1,023.65 (483.54 to 1,563.76)	<.001
2019	607.73 (293.53 to 921.93)	<.001
2020	783.24 (228.12 to 1,338.37)	.006
2021	1,090.21 (300.40 to 1,880.02)	.007
**Emergency department visits**		
Pooled	151.52 (101.76 to 201.27)	<.001
2016	160.21 (63.96 to 256.46)	.001
2017	215.70 (108.55 to 322.85)	<.001
2018	129.54 (30.72 to 228.36)	.01
2019	158.20 (58.30 to 258.10)	.002
2020	135.12 (17.17 to 253.08)	.03
2021	78.97 (18.43 to 139.51)	.01
**Hospital inpatient stays**		
Pooled	685.39 (291.11 to 1,079.67)	.001
2016	349.50 (−228.45 to 927.45)	.24
2017	962.20 (−124.84 to 2,049.24)	.08
2018	838.26 (146.63 to 1,529.89)	.02
2019	833.57 (65.12 to 1,602.02)	.03
2020	1,530.18 (−639.16 to 3,699.52)	.17
2021	295.25 (−260.48 to 850.98)	.30
**Hospital outpatient**		
Pooled	243.22 (66.15 to 420.30)	.007
2016	164.73 (35.64 to 293.82)	.01
2017	221.02 (4.32 to 437.72)	.046
2018	383.09 (42.63 to 723.55)	.03
2019	108.36 (−79.77 to 296.49)	.26
2020	223.55 (−163.00 to 610.09)	.26
2021	257.25 (−20.28 to 534.79)	.07
**Home health**		
Pooled	99.34 (−5.21 to 203.90)	.06
2016	320.37 (−29.94 to 670.69)	.07
2017	−28.32 (−146.42 to 89.79)	.64
2018	153.39 (−142.27 to 449.06)	.31
2019	197.39 (−79.06 to 473.84)	.16
2020	118.43 (−134.77 to 371.64)	.36
2021	329.55 (−498.06 to 1,157.16)	.43

^a^ Data source: 2016–2021 Medical Expenditures Panel Survey. All estimates were adjusted for the sociodemographic factors included in the analysis. All estimates are pooled and weighted, adjusting for the complex survey design. Expenditures are adjusted to 2021 US dollars.

^b^ Determined by Pearson χ^2^ test.

We found differences in total medical expenditures, emergency department visits, office-based medical visits, and prescribed medicines expenditures by treated asthma status for each race and ethnicity (Figure). Black children with treated asthma had $2,721.28 lower total medical expenditures and $803.19 lower office-based medical visit expenditures than White children with treated asthma.

**Figure:**
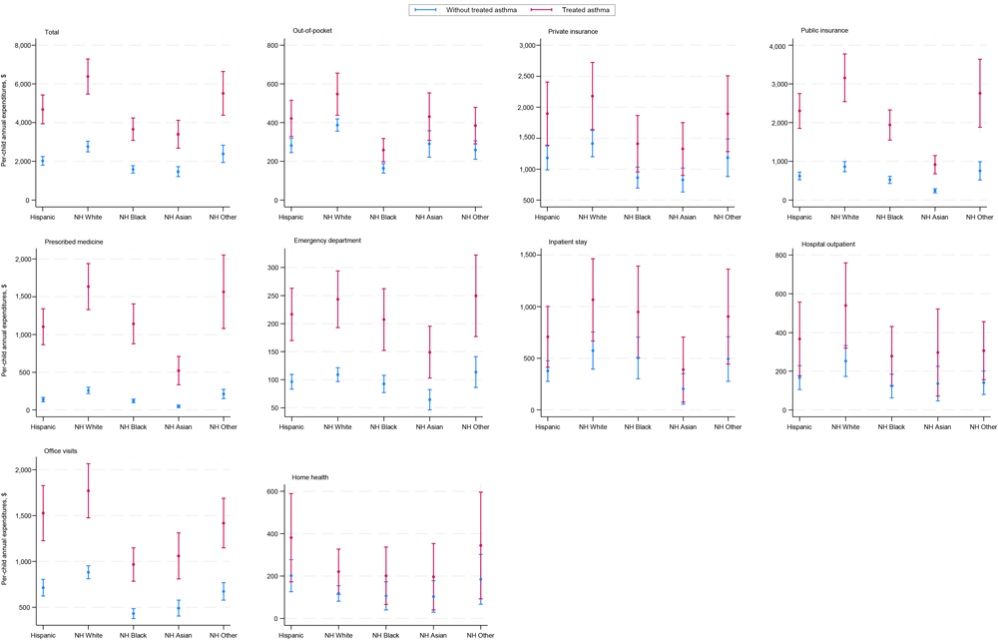
Total medical expenditures among children and adolescents aged 0 to 17 years, in dollars, by treated asthma and race and ethnicity, 2016–2021 Medical Expenditures Panel Survey. All estimates were pooled and weighted using the complex survey design. Non-Hispanic Other race includes American Indian, Alaska Native, and multiracial. Graphs use different scales. Abbreviation: NH, non-Hispanic.

**Table:** 

Characteristic	Estimate (95% CI), $
**Total**	
Hispanic without asthma	2,029.38 (1,800.36–2,258.41)
Non-Hispanic White without asthma	2,766.22 (2,490.28–3,042.16)
Non-Hispanic Black without asthma	1,587.32 (1,395.82–1,778.81)
Non-Hispanic Asian without asthma	1,474.32 (1,215.68–1,732.96)
Non-Hispanic Other without asthma	2,390.16 (1,943.85–2,836.47)
Hispanic with asthma	4,684.44 (3,939.50–5,429.39)
Non-Hispanic White with asthma	6,385.30 (5,483.77–7,286.82)
Non-Hispanic Black with asthma	3,664.02 (3,086.90–4,241.14)
Non-Hispanic Asian with asthma	3,403.19 (2,678.69–4,127.69)
Non-Hispanic Other with asthma	5,517.24 (4,389.73–6,644.75)
**Out-of-pocket**	
Hispanic without asthma	282.26 (245.48–319.04)
Non-Hispanic White without asthma	387.90 (356.07–419.73)
Non-Hispanic Black without asthma	164.34 (140.28–188.40)
Non-Hispanic Asian without asthma	290.78 (222.35–359.22)
Non-Hispanic Other without asthma	259.13 (211.58–306.68)
Hispanic with asthma	421.89 (327.87–515.91)
Non-Hispanic White with asthma	547.73 (438.53–656.93)
Non-Hispanic Black with asthma	258.62 (198.62–318.62)
Non-Hispanic Asian with asthma	431.34 (309.06–553.61)
Non-Hispanic Other with asthma	385.28 (291.34–479.23)
**Private insurance**	
Hispanic without asthma	1,182.54 (989.16–1,375.91)
Non-Hispanic White without asthma	1,413.44 (1,199.93–1,626.96)
Non-Hispanic Black without asthma	863.91 (694.04–1,033.77)
Non-Hispanic Asian without asthma	825.94 (632.88–1,019.00)
Non-Hispanic Other without asthma	1,185.43 (882.10–1,488.76)
Hispanic with asthma	1,896.83 (1,385.54–2,408.13)
Non-Hispanic White with asthma	2,180.08 (1,638.08–2,722.07)
Non-Hispanic Black with asthma	1,410.99 (954.84–1,867.13)
Non-Hispanic Asian with asthma	1,326.31 (900.69–1,751.93)
Non-Hispanic Other with asthma	1,894.63 (1,280.94–2,508.32)
**Public insurance**	
Hispanic without asthma	623.90 (525.13–722.67)
Non-Hispanic White without asthma	863.02 (731.89–994.15)
Non-Hispanic Black without asthma	524.47 (431.83–617.11)
Non-Hispanic Asian without asthma	239.41 (184.50–294.32)
Non-Hispanic Other without asthma	756.93 (521.53–992.32)
Hispanic with asthma	2,305.29 (1,856.67–2,753.91)
Non-Hispanic White with asthma	3,158.39 (2,541.39–3,775.39)
Non-Hispanic Black with asthma	1,941.63 (1,552.53–2,330.73)
Non-Hispanic Asian with asthma	916.63 (683.60–1,149.67)
Non-Hispanic Other with asthma	2,759.74 (1,881.55–3,637.93)
**Prescribed medicine**	
Hispanic without asthma	137.15 (109.11–165.19)
Non-Hispanic White without asthma	258.53 (217.52–299.53)
Non-Hispanic Black without asthma	120.65 (95.42–145.87)
Non-Hispanic Asian without asthma	47.55 (30.40–64.69)
Non-Hispanic Other without asthma	211.84 (151.35–272.34)
Hispanic with asthma	1,103.57 (866.94–1,340.19)
Non-Hispanic White with asthma	1,635.33 (1,329.92–1,940.73)
Non-Hispanic Black with asthma	1,141.20 (877.38–1,405.02)
Non-Hispanic Asian with asthma	521.43 (333.61–709.24)
Non-Hispanic Other with asthma	1,564.69 (1,077.53–2,051.84)
**Emergency room**	
Hispanic without asthma	96.42 (83.35–109.49)
Non-Hispanic White without asthma	109.16 (96.94–121.39)
Non-Hispanic Black without asthma	92.51 (77.13–107.90)
Non-Hispanic Asian without asthma	64.29 (46.11–82.48)
Non-Hispanic Other without asthma	113.66 (86.29–141.02)
Hispanic with asthma	216.50 (169.89–263.11)
Non-Hispanic White with asthma	243.29 (192.74–293.84)
Non-Hispanic Black with asthma	207.12 (152.26–261.99)
Non-Hispanic Asian with asthma	149.05 (102.98–195.12)
Non-Hispanic Other with asthma	249.43 (176.92–321.94)
**Inpatient stay**	
Hispanic without asthma	377.68 (277.75–477.61)
Non-Hispanic White without asthma	575.02 (395.60–754.43)
Non-Hispanic Black without asthma	504.67 (303.00–706.34)
Non-Hispanic Asian without asthma	204.37 (56.25–352.48)
Non-Hispanic Other without asthma	494.21 (278.56–709.85)
Hispanic with asthma	708.37 (413.76–1,002.98)
Non-Hispanic White with asthma	1,067.26 (668.54–1,465.98)
Non-Hispanic Black with asthma	949.40 (506.18–1,392.61)
Non-Hispanic Asian with asthma	391.08 (76.50–705.67)
Non-Hispanic Other with asthma	905.00 (444.51–1,365.50)
**Hospital outpatient**	
Hispanic without asthma	167.08 (104.94–229.22)
Non-Hispanic White without asthma	252.49 (172.78–332.21)
Non-Hispanic Black without asthma	123.84 (62.43–185.25)
Non-Hispanic Asian without asthma	136.27 (46.99–225.54)
Non-Hispanic Other without asthma	141.03 (80.42–201.63)
Hispanic with asthma	366.81 (176.37–557.25)
Non-Hispanic White with asthma	539.94 (320.09–759.78)
Non-Hispanic Black with asthma	278.25 (125.07–431.43)
Non-Hispanic Asian with asthma	297.05 (71.78–522.31)
Non-Hispanic Other with asthma	306.07 (156.04–456.11)
**Office visits**	
Hispanic without asthma	714.03 (624.33–803.73)
Non-Hispanic White without asthma	882.43 (811.71–953.15)
Non-Hispanic Black without asthma	432.98 (379.03–486.94)
Non-Hispanic Asian without asthma	491.88 (405.71–578.04)
Non-Hispanic Other without asthma	673.55 (577.73–769.36)
Hispanic with asthma	1,526.21 (1,227.06–1,825.36)
Non-Hispanic White with asthma	1,769.65 (1,473.58–2065.72)
Non-Hispanic Black with asthma	966.46 (783.46–1,149.46)
Non-Hispanic Asian with asthma	1,059.73 (808.57–1,310.89)
Non-Hispanic Other with asthma	1,417.75 (1,147.82–1,687.69)
**Home health visits**	
Hispanic without asthma	201.61 (126.27–276.94)
Non-Hispanic White without asthma	117.92 (81.51–154.34)
Non-Hispanic Black without asthma	106.20 (40.39–172.00)
Non-Hispanic Asian without asthma	103.49 (29.34–177.64)
Non-Hispanic Other without asthma	184.66 (67.45–301.88)
Hispanic with asthma	380.77 (172.60–588.95)
Non-Hispanic White with asthma	220.78 (114.31–327.25)
Non-Hispanic Black with asthma	201.29 (66.40–336.19)
Non-Hispanic Asian with asthma	196.17 (39.94–352.41)
Non-Hispanic Other with asthma	343.68 (92.40–594.96)

## Discussion

In this study of US children from 2016 through 2021, we found that children with treated asthma had significantly higher medical expenditures ($3,362.56 in 2021 US dollars) than children without treated asthma. Previous research of 2008-2013 MEPS data found an average of $3,266 in incremental medical expenditures attributed to asthma for the total population and $1,737 in 2015 US dollars for children (4). Our incremental medical expenditure estimates for children with treated asthma are higher than previous incremental medical expenditure estimates for all ages of treated asthma, which may suggest that the incremental medical expenditures of treated asthma for children have been increasing over time.

We found that the incremental medical costs of US children with asthma from 2016 through 2021 included $955.96 for prescribed medicines, $151.52 for emergency department visits, $858.17 for office-based medical visits, $685.39 for inpatient hospital stays, $243.22 in hospital-based outpatient visits, and $99.34 in home health visits. Previous estimates of the total US population that used data from the 2008–2013 MEPS found incremental medical expenditures of $1,830 in prescribed medicines, $105 in emergency department visits, $640 in office-based medical visits, $529 in inpatient hospital stays, and $176 in hospital-based outpatient visits for people with asthma (4). The incremental expenditures estimates based on the 2008–2013 MEPS data were smaller than the estimates in our study for every category except for prescribed medicines, which might be explained by children typically having less use of prescribed medicines than the population of all ages (15). However, in the 2008–2013 MEPS analysis, US children had significantly lower total incremental medical expenditures for asthma compared with adults ($1,737 vs $3,761). In addition, the 2008–2013 MEPS children’s incremental expenditure estimate was lower than the $3,362.56 of total incremental expenditures of treated asthma for children in our study, which was based on more recent (2016–2021) data. Another study of US school-aged children (aged 6–17 y) in 2007–2013 found incremental costs of $847 in total medical expenditures, $360 in prescribed medicines, and $132 in emergency department visits due to asthma (16). Our results might reflect increases in the use and/or costs of medical events for children who seek medical treatment for asthma in recent years. Future research can be conducted to estimate the causes of medical use and cost trends for asthma.

Prescribed medicines were a large component of medical expenditures for children with treated asthma. The cost of asthma medication increased by 36% from 2013 to 2018, from $280 to $380 (17). Because we estimated the expenditures of all prescribed medicines and not just asthma medicine, it is possible that children with treated asthma were also taking medicine for other comorbid conditions, as previous research shows higher prevalences of almost all comorbidities for children with asthma compared with children without asthma (18). Additional analyses can examine the types of medicines prescribed for children with asthma and their implications for the cost of treating asthma.

Consistent with previous results, our findings indicate that Black and Hispanic children have a significantly higher prevalence of treated asthma than White children (2). Previous research showed that the asthma burden is greater among non-Hispanic Black children than non-Hispanic White children even after adjusting for confounding factors of demographic characteristics, socioeconomic status, comorbidities, and asthma treatment compliance (19). At the same time, our study showed that Black and Hispanic children with treated asthma had lower total medical expenditures than White children with treated asthma. This matches the patterns of racial and ethnic disparity seen in patients with diabetes (20) and may be due to the reduced use of health services and medication by Black and Hispanic children. Our analysis by medical event showed no significant differences between Black and White children in medical event expenditures, except for office-based visits. Previous research showed that non-Hispanic Black children with asthma had fewer family provider visits for asthma and were less likely to receive a written asthma treatment plan from their health care provider than non-Hispanic White children with asthma (21). Also, previous research showed that Black children with asthma had the lowest likelihood of using the physician’s office as the usual place of care among children of all racial and ethnic groups with asthma (22). Research shows heightened medical mistrust among non-Hispanic Black patients compared with non-Hispanic White patients as a reason for reduced primary care use (23). Historical events such as the Tuskegee syphilis study are often cited as reasons for heightened medical mistrust among Black persons (24). Further research can improve our understanding of racial disparities in seeking asthma treatment through physicians’ offices.

In our analysis, the prevalence of treated asthma among non-Hispanic Asian children was not significantly different from the prevalence among non-Hispanic White children, but non-Hispanic Asian children had lower medical expenditures than non-Hispanic White children. Previous research based on the 2002–2008 MEPS data showed that non-Hispanic Asian persons had lower medical expenditures than non-Hispanic White persons (25). Another study of 2013–2016 MEPS data showed differences in medical expenditures among Asian subgroups, while Asian persons had lower medical expenditures overall compared with non-Hispanic White persons (26). Both studies suggested that differences in medical expenditures were due to differences in nativity (non–US-born vs US-born) and English language proficiency between non-Hispanic Asian and non-Hispanic White children and families.

We did not find significant disparities in treated asthma prevalence based on family income. This finding matches research that concluded that racial disparities in childhood asthma are not solely the result of socioeconomic disparities between racial groups, because race is a complex construct of genetic, environmental, and social factors (27). Historical practices such as redlining, which classified neighborhoods with higher concentrations of Black and other racial minorities as less financially desirable, led to racial and socioeconomic segregation and are associated with present-day disparities in asthma prevalence and severity (28).

Our study period coincided with the COVID-19 pandemic. The prevalence of treated asthma decreased during 2020 and 2021; this decrease may be attributed to individuals avoiding nonurgent medical services during the pandemic and the decreased availability of outpatient medical visits. Telehealth became more available during the COVID-19 pandemic; however, this increased availability could have affected health care delivery among patients who did not have access to telehealth technology or did not have insurance coverage for telehealth services (29). Previous research found that medication adherence increased during the COVID-19 pandemic, which might have increased expenditures for prescribed medicines (29). Also, emergency department visits for pediatric asthma decreased in the early months of 2020 (29). Additional research on the costs of treating asthma during the COVID-19 pandemic and the role of telehealth can reveal the costs and benefits of telehealth.

Our study found lower average total and out-of-pocket medical expenditures among Black and Hispanic children with treated asthma than among White children with treated asthma. However, this finding does not mean that the economic burden was reduced among Black and Hispanic families. For example, previous research found that parents of Black and Hispanic children were less likely to have annual out-of-pocket medical expenditures of more than $1,000, but they were more likely to report unreasonably high out-of-pocket spending than the parents of White children (30).

We found that out-of-pocket medical expenditures for children with treated asthma were not significantly different than those for children without treated asthma in 2020 and 2021. This finding suggests a decrease in the use of medical events that contributed to increased out-of-pocket expenditures for children with treated asthma or a decrease in out-of-pocket expenditure during these years. Further analysis can examine the types of medical events that contribute to disparities in out-of-pocket medical expenditures between children with and without treated asthma.

We found that public payers generally incurred the highest incremental medical expenditures for asthma among US children, followed by private payers. Children with treated asthma were more likely than children without treated asthma to have public health insurance, which means that public health insurance coverage of asthma care is crucial to eliminating inequities in health expenditures (31). Previous analysis of data from the 2012–2014 Child Asthma Call-Back Survey found that public insurance, compared with private insurance, was associated with cost barriers to seeking a physician for care (32). A study of children who were hospitalized for asthma and had public insurance found that these children had longer stays, higher costs, and higher readmission odds compared with privately insured children (33). Further research can examine the interaction of the types of health insurance and the types of health services that are covered for asthma care.

### Limitations

This study has several limitations. First, our data did not assess all the various social determinants of health, such as the built environment, that can affect exposure to indoor and outdoor asthma triggers (34). Second, we did not assess the severity of asthma, which can affect the medical costs of treated asthma (35). Third, we did not examine the total economic burden of asthma among children, which includes days absent from school and was considered in previous research (4). However, after 2016, the MEPS no longer collected data on school absenteeism. Therefore, we could not measure the costs of missed school days due to asthma among children.

### Conclusion

Childhood asthma remains a substantial health and economic burden for US families, payers, providers, and the overall society. The incremental costs of treated asthma for children from 2016 through 2021 were higher than in previous estimates, which suggests an urgent need to promote and implement cost-effective asthma control programs. Black and Hispanic children have a higher prevalence of treated asthma than White children, but not necessarily higher medical expenditures. This finding might be driven by disparities in office-based medical visit expenditures and by possible undertreatment of asthma. Further investigation of how to improve access to and the quality of asthma care for disproportionately affected children can help to advance health equity in asthma prevalence and asthma-related medical expenditures.
